# A universal protocol for high-quality DNA and RNA isolation from diverse plant species

**DOI:** 10.1371/journal.pone.0295852

**Published:** 2023-12-14

**Authors:** Farhad Masoomi-Aladizgeh, Leila Jabbari, Reza Khayam Nekouei, Ali Aalami, Brian J. Atwell, Paul A. Haynes

**Affiliations:** 1 School of Natural Sciences, Macquarie University, Sydney, NSW, Australia; 2 Department of Tissue Culture and Gene Transformation, Agricultural Biotechnology Research Institute of Iran (ABRII), AREEO, Karaj, Iran; 3 Department of Agronomy and Plant Breeding, Faculty of Agricultural Sciences, University of Guilan, Rasht, Iran; Hainan University, CHINA

## Abstract

Next-generation sequencing demands high-quality nucleic acid, yet isolating DNA and RNA is often challenging, particularly from plant tissues. Despite advances in developing various kits and reagents, these products are tailored to isolation of nucleic acid from model plant tissues. Here we introduce a universal lysis buffer to separate nucleic acid from various plant species, including recalcitrant plants, to facilitate molecular analyses, such as quantitative PCR (qPCR), transcriptomics, and whole-genome sequencing (WGS). The protocol is a modification of the original CTAB methods, which leads to nucleic acid isolation from many plant species, including monocots and eudicots. The lysis buffer consists of hexadecyltrimethylammonium bromide (CTAB), sodium chloride (NaCl), Tris base, ethylenediaminetetraacetic acid (EDTA) and β-mercaptoethanol (βME). The modified CTAB method enables the isolation of nucleic acid from small amounts of plant tissues (e.g., 15–100 mg) in a timely manner, which is well-suited for a large number of samples and also when adequate sample collection is a limiting factor. The protocol isolates not only DNA from various plant species but also RNA. This makes it highly effective for molecular analyses compared to previously described CTAB methods optimised for DNA isolation. The appropriate concentration of the components enables high-quality DNA and RNA isolation from plant tissues simultaneously. Additionally, this protocol is compatible with commercially available columns. For DNA and RNA to be qualified for next-generation sequencing platforms, the protocol is supplemented with columns to purify either DNA or RNA from the same tissue to meet high standards for sequencing analyses. This protocol provides an ideal approach to overcome potential obstacles in isolating high-quality DNA or RNA from a wide range of plant species for downstream molecular analysis.

## Introduction

Studying nucleic acid commenced with Johannes Friedrich Miescher in 1869, who identified a substance with unexpected properties called *nuclein* (today called DNA) [1]. To conduct molecular studies, nucleic acid, including DNA and RNA, must be isolated from biological samples. Isolating high-quality nucleic acid is a critical step for downstream molecular analysis, yet it is challenging for many non-model plant species.

There are many protocols which have been developed to isolate nucleic acid from plant species. One widely used protocol for plant DNA isolation is the cetyltrimethylammonium bromide (CTAB) protocol developed by Murray and Thompson (1980) [2] and Saghai-Maroof et al. (1984) [3]. This protocol was later optimised to isolate DNA from various plant tissues by Doyle and Doyle (1987) [4]. Since then, the CTAB protocol has been modified thoroughly to enable the isolation of DNA from various plant species, due to their unique secondary metabolites [5]. For example, Allen et al. (2006) optimised the CTAB protocol to isolate DNA from plant tissues, by using less expensive and toxic reagents [6]. Similarly, there have been improvements regarding RNA isolation protocols from plant tissues. For example, Chomczynski and Sacchi (2006) developed a single-step method based on acid guanidinium thiocyanate-phenol-chloroform, which can be universally used for RNA extraction from different biological samples [7]. Later, Ghawana et al. (2011) described an RNA isolation protocol suitable for plant tissues rich in secondary metabolites [8]. Many other protocols have been developed for isolating nucleic acid from plant tissues, which are often modified versions of original protocols.

Since plant species are diverse, no universal protocol has been described to isolate nucleic acid from a wide range of species. Researchers usually test several protocols or purchase commercial reagents and kits to obtain high-quality DNA and RNA, which is time-consuming and expensive. Moreover, isolating high-quality nucleic acid from plants rich in polysaccharide and polyphenolic compounds is challenging. In fact, low-quality DNA and RNA can compromise downstream molecular analysis, such as next-generation sequencing.

Here, a universal protocol facilitating the isolation of high-quality DNA and RNA from diverse plant species is described. The protocol presented here is the modified CTAB protocol, which enables the isolation of not only DNA from various plant species but also RNA from the same tissues. The lysis buffer described in this protocol consists of 0.5% CTAB, 1% EDTA, 2.5% Tris base, 5% NaCl and 5% β-mercaptoethanol (βME), and has been demonstrated on separation of DNA and RNA from a wide range of plant species. The appropriate concentration of the components creates an ideal pH, which then facilitates the isolation of DNA and RNA from plant tissues simultaneously. This is one of the key differences between this protocol and other CTAB protocols that use similar reagents but have been optimised to isolate only DNA. This protocol has been used by independent researchers, demonstrating its reliability and reproducibility. We describe in detail how the modified CTAB method can also be used for RNA isolation. Two procedures are introduced here, ‘solution-based’ and ‘column-based’, resulting in high-quality DNA or RNA. The latter procedure is recommended for next-generation sequencing, such as whole-genome sequencing (WGS) and RNA-seq analyses. The protocol is ideal for researchers experiencing difficulties isolating high-quality nucleic acid from plant tissues, especially recalcitrant species, using commercial kits, reagents and original CTAB methods.

## Materials and methods

### 1. Lysis buffer for DNA and RNA isolation

**Fig 1:**
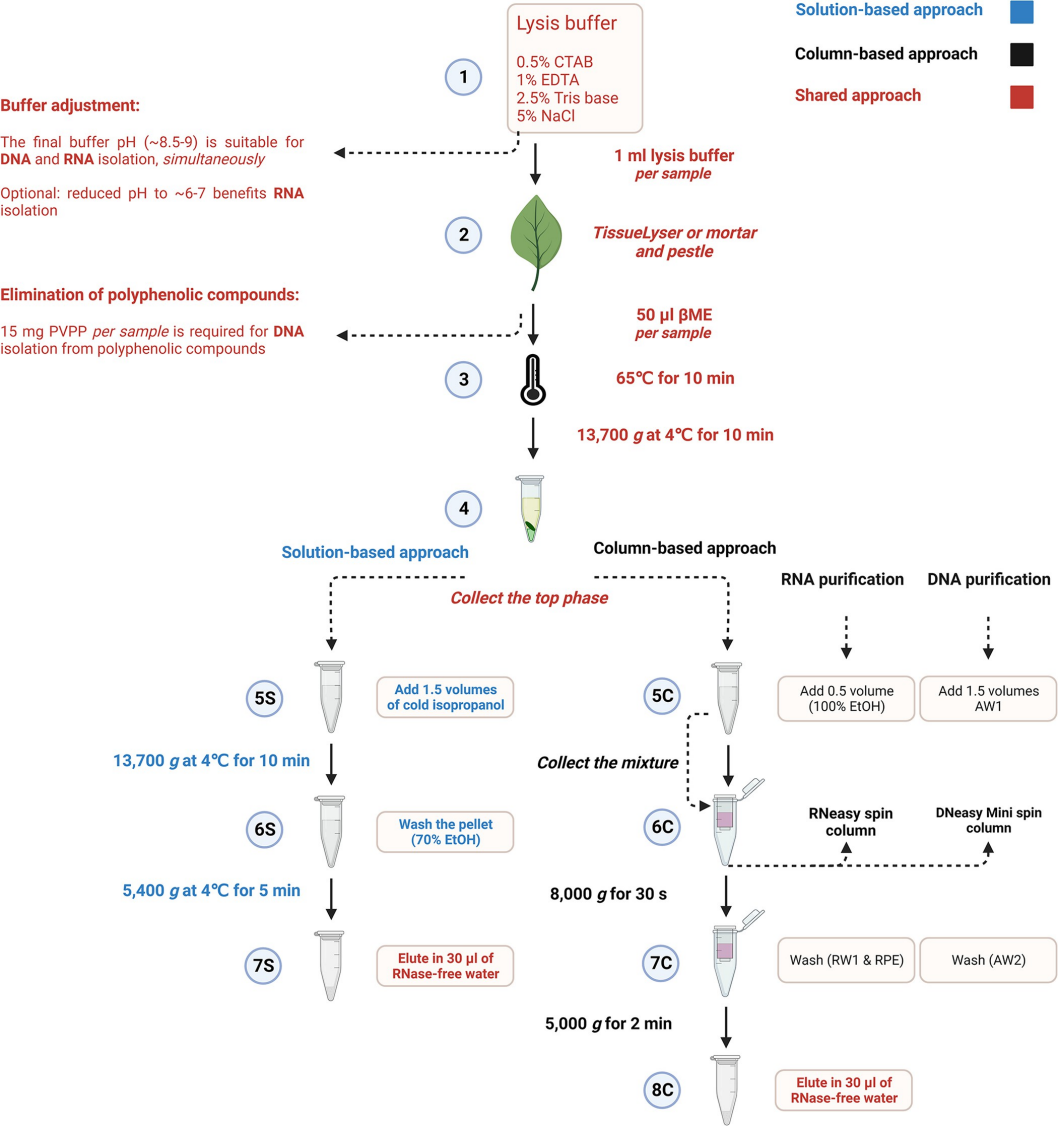
Nucleic acid isolation from plant species. Steps 1–4 include the preparation of lysis buffer for DNA and RNA isolation (Step 1), homogenisation of tissues (Step 2), lysis of tissues (Step 3) and triple-phase separation (Step 4), the main steps of nucleic acid isolation. ***Optional step*:** *The pH of the lysis buffer is at approximately 8*.*5–9*, *simultaneously isolating DNA and RNA from plant tissues*. *Reduce the pH of the lysis buffer to approximately 6–7 to isolate only RNA*. The upper phase, containing nucleic acid collected from Step 4, can be purified either using the solution-based approach (5S-7S) or the column-based approach (5C-8C). **Solution-based approach:** Collect the upper phase from Step 4 and mix it with 1.5 volumes of cold isopropanol (Step 5S). Centrifuge the mixture at 13,700 *g* at 4°C for 10 min. Discard the supernatant and wash the pellet gently with 70% EtOH (Step 6S), followed by centrifugation at 5,400 *g* at 4°C for 5 min. Remove the supernatant, and then air dry the pellet. Resuspend the pellet in 30 μl of RNase-free or autoclaved water by incubating at room temperature (Step 7S). **Column-based approach for RNA:** Collect the upper phase from Step 4, add 0.5 volume of EtOH (100%) to the lysate, and mix it immediately by pipetting (Step 5C). Transfer the mixture into an RNeasy spin column (pink), including any precipitate (Step 6C). Centrifuge the mixture at 8,000 *g* for 30 s, and discard the flow-through. Place the RNeasy spin column into a new tube, add buffers RW1 (700 μl) and then RPE (500 μl) to the RNeasy spin column, followed by centrifugation each time at 8,000 *g* for 30 s to wash the spin column membrane (Step 7C). Transfer the RNeasy spin column to a new tube, and add 30 μl RNAse-free water directly onto the RNeasy membrane. Centrifuge the samples at 5,000 *g* for 2 min to elute RNA (Step 8C). **Column-based approach for DNA:** Collect the upper phase from Step 4, add 1.5 volumes of buffer AW1 to the lysate, and mix it immediately by pipetting (Step 5C). Transfer the mixture into a DNeasy Mini spin column (white), including any precipitate (Step 6C). Centrifuge the mixture at 8,000 *g* for 30 s, and discard the flow-through. Place the DNeasy Mini spin column into a new tube, add buffer AW2 (500 μl) to the DNeasy spin column, followed by centrifugation at 8,000 *g* for 30 s to wash the spin column membrane (Step 7C). Transfer the DNeasy Mini spin column into a new tube, and add 30 μl RNAse-free water directly onto the DNeasy membrane. Centrifuge the samples at 5,000 *g* for 2 min to elute DNA (Step 8C).

The lysis buffer is developed using 0.5% CTAB (Sigma-Aldrich), 1% EDTA (Sigma-Aldrich), 2.5% Tris base (Sigma-Aldrich) and 5% NaCl (Astral Scientific) for DNA and RNA isolation from plant tissues. The ratio of the components maintains the pH of the lysis buffer at approximately 8.5–9, which is effective for simultaneously isolating DNA and RNA from the mixture. The pH of the lysis buffer can be reduced to approximately 6–7 to precipitate DNA into the organic phase and enable the isolation of pure RNA from the mixture (Fig 1, Step 1).

### 2. Homogenisation of tissues

Plant tissues are ground (e.g. leaf, shoot, root, approximately 0.1 g) with 1 ml of the lysis buffer (0.5% CTAB, 1% EDTA, 2.5% Tris base and 5% NaCl) in a sterilised mortar and pestle, without liquid nitrogen. Alternatively, the tissues are ground, without the lysis buffer, with a TissueLyser (TissueLyser II, Qiagen) using up to six Zirconox beads (2.8–3.3 mm) and liquid nitrogen. The ground tissues are then mixed with 1 ml of the lysis buffer (Fig 1, Step 2).

### 3. Lysis of tissues

The ground tissues with 1 ml of the lysis buffer are vigorously vortexed to create a homogeneous mixture. For DNA and RNA isolation, 50 μl (5%) βME (Sigma-Aldrich) is freshly added to the mixture to decrease the probable adventitious oxidation, especially for tissues with high polysaccharides and secondary metabolites. βME is highly recommended for RNA isolation since it eliminates RNAses released during cell lysis. For DNA isolation, 15 mg (1.5%) polyvinylpolypyrrolidone (PVPP, Sigma-Aldrich) is added to 1 ml of lysis buffer to increase the concentration of DNA, only for tissues with high levels of polyphenolic compounds. Then, the samples are incubated in a water bath at 65°C for 10 min to lyse cells completely. The mixture is inverted several times during the incubation time. Alternatively, a shaker incubator is used to incubate the samples at 65°C for 10 min with constant shaking (Fig 1, Step 3).

### 4. Triple-phase separation

A total of 600 μl chloroform (Sigma-Aldrich) is added to the lysate. The lysate is then vortexed vigorously, followed by centrifugation at 13,700 *g* at 4°C for 10 min, to facilitate the separation of three phases, including aqueous phase (top), followed by solid phase (middle) and organic phase (bottom). The upper aqueous phase (600 μl) is transferred to a new 2 ml microcentrifuge tube gently, without disrupting the solid and organic phases. This phase is then suitable for solution-based or column-based approaches for nucleic acid purification and precipitation (Fig 1, Step 4).

### 5. Purification and precipitation of nucleic acid

#### 5.1 Solution-based approach

For DNA and RNA isolation using the solution-based approach, the upper aqueous phase (600 μl) is mixed with 1.5 volumes (900 μl) of cold isopropanol (Sigma-Aldrich, Fig 1, Step 5S). The mixture is centrifuged at 13,700 *g* at 4°C for 10 min. The supernatant is discarded, and the pellet is washed gently with 70% EtOH (Chem-Supply Pty Ltd Australia, Fig 1, Step 6S). Then, the mixture is centrifuged at 5,400 *g* at 4°C for 5 min, the supernatant is discarded, and the pellet is air dried. The pellet is resuspended in 30 μl of RNase-free or autoclaved water (Fig 1, Step 7S).

#### 5.2 Column-based approach

*5*.*2*.*1 RNA isolation (coupled with the Qiagen RNeasy Plant Mini Kit)*. For RNA isolation using the column-based approach, the upper aqueous phase (600 μl) is mixed with 0.5 volume of EtOH (100%, 300 μl) (Fig 1, Step 5C). The mixture, including any precipitate, is transferred into an RNeasy spin column (pink) (Fig 1, Step 6C), and purified following the manufacturer’s instructions (Qiagen, Hilden, Germany). Briefly, the mixture is centrifuged at 8,000 *g* for 30 s, and the flow-through is discarded. The buffers RW1 (700 μl) and then RPE (500 μl) are added separately to the spin column, followed by centrifugation each time at 8,000 *g* for 30 s (Fig 1, Step 7C). RNAse-free water (30 μl) is directly added onto the RNeasy membrane, followed by centrifugation at 5,000 *g* for 2 min to elute RNA (Fig 1, Step 8C).

*5*.*2*.*2 DNA isolation (coupled with the Qiagen DNeasy Plant Mini Kit)*. For DNA isolation using the column-based approach, the upper aqueous phase (600 μl) is mixed with 1.5 volumes of the buffer AW1 (900 μl) (Fig 1, Step 5C), and purified following the manufacturer’s instructions (Qiagen, Hilden, Germany). Briefly, the mixture, including any precipitate, is transferred into a DNeasy Mini spin column (white) (Fig 1, Step 6C). The mixture is centrifuged at 8,000 *g* for 30 s, and the flow-through is discarded. The buffer AW2 (500 μl) is added to the spin column, followed by centrifugation at 8,000 *g* for 30 s (Fig 1, Step 7C). RNAse-free water (30 μl) is directly added onto the DNeasy membrane, followed by centrifugation at 5,000 *g* for 2 min to elute DNA (Fig 1, Step 8C). Fig 1 demonstrates the workflow of DNA and RNA isolation following solution-based and column-based approaches. This protocol described in this peer-reviewed article is published on protocols.io in detail, https://dx.doi.org/10.17504/protocols.io.8epv5xj86g1b/v3 and is included for printing as S1 File with this article.

## Expected results

Many methods have been developed to isolate nucleic acid from plant tissues, which are often time-consuming, costly and not widely applicable. The great diversity of plant species increases the complexity of nucleic acid isolation; this is mainly due to the high content of compounds such as secondary metabolites, polyphenols, and polysaccharides in plant tissues. These compounds interfere with nucleic acid isolation and purification, adversely affecting downstream molecular analysis.

We developed a straightforward protocol enabling the isolation of high-quality nucleic acid from various plant species. The following four steps were commonly used in the protocol for DNA and RNA isolation, using either solution-based or column-based approaches. These steps included the preparation of lysis buffer for DNA and RNA isolation (Fig 1, Step 1), homogenisation of tissues (Fig 1, Step 2), lysis of tissues (Fig 1, Step 3) and triple-phase separation (Fig 1, Step 4). These steps resulted in isolating a high concentration of DNA and RNA from plant tissues, followed by purification of nucleic acids using solution-based (Fig 1, Steps 5S-7S) and column-based (Fig 1, Steps 5C-8C) approaches. Depending on the purpose of the research, either approach can be used for nucleic acid purification. These approaches have been successfully used for Southern blotting, quantitative PCR (qPCR), Sanger sequencing and Next Generation Sequencing (NGS) analyses. The column-based approach has been demonstrated to isolate high-quality DNA and RNA, which are suitable for WGS and RNA-seq analyses.

The protocol described here successfully isolated nucleic acid from various plant species, including recalcitrant species. The tested plant species include *Oryza sativa*, *Hordeum vulgare*, *Brassica napus*, *Juglans regia*, *Malus domestica*, *Solanum tuberosum* L., *Thymus vulgaris*, *Pelargonium* spp. [9], olive cultivars [10], *Malus pumila* Mill. [11], *Pyrus communis* L. [12], *Pyrus* L. [13], genus *Triticum* (including 12 species) [14–16], *Gossypium robinsonii* [17], *Gossypium hirsutum* [18], *Prunus persica* L. [19], *Cydonia oblonga* Mill. [20], *Themeda triandra* [21], demonstrating a broad application of this protocol and the reproducibility of the outcome. Moreover, it has been shown that the protocol enables isolation of nucleic acid from non-plant species such as bacterial strains [22], mites (*Amblyseius swirskii* [23] and *Typhlodromus bagdasarjani* [24]) and even the model yeast (*Saccharomyces cerevisiae*), after disrupting cell wall with glass beads and 20 mM NaOH.

The quality of DNA isolated using the protocol was validated by the Beijing Genomics Institute (https://www.bgi.com/global) prior to WGS (short-read sequencing) analysis [17], while the quality of RNA isolated using the protocol was validated by the Ramaciotti Centre for Genomics (https://www.ramaciotti.unsw.edu.au/) prior to RNA-seq analysis [18].

**Fig 2:**
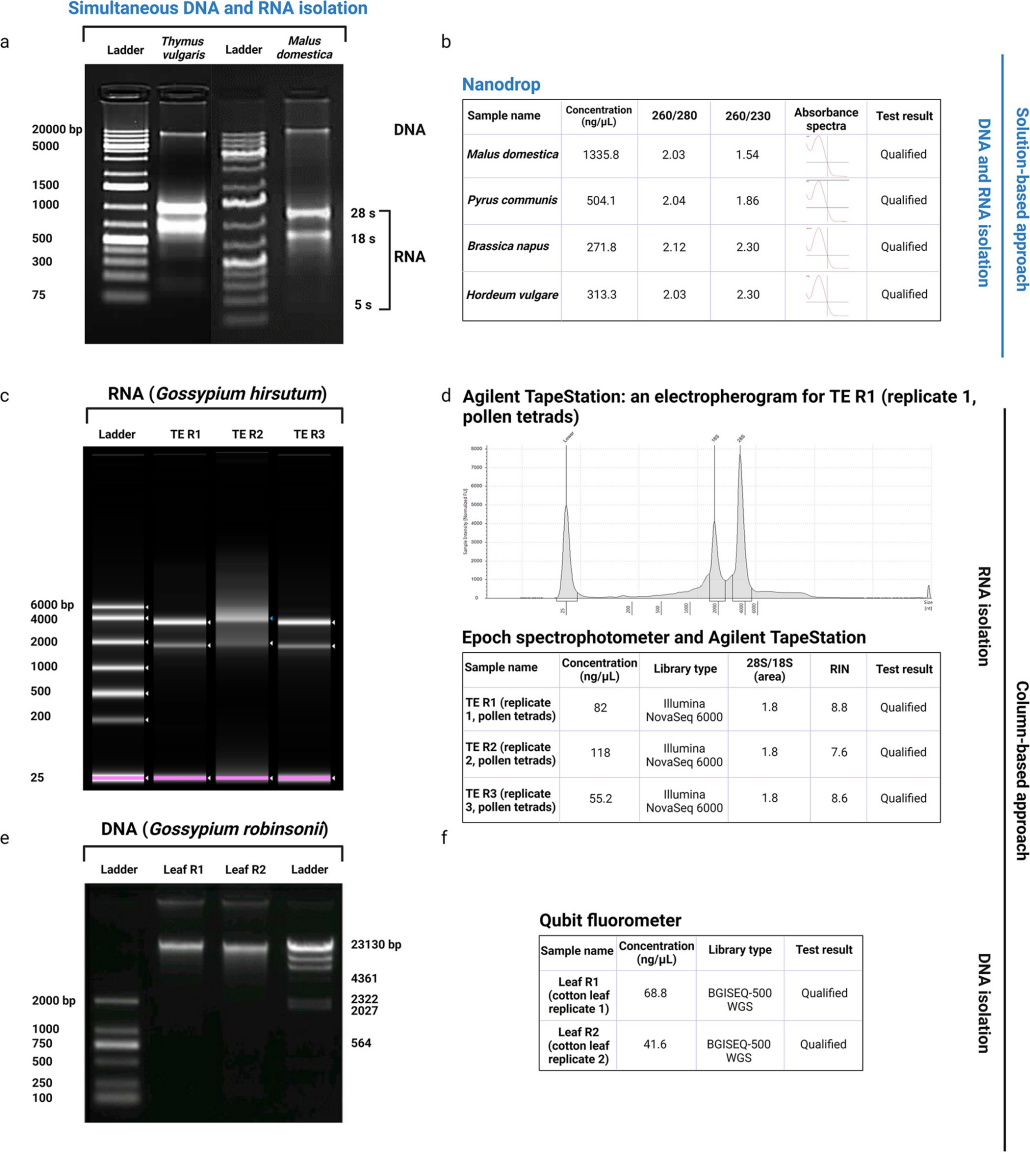
Quality and quantity of nucleic acid isolation using solution-based and column-based approaches. (a) DNA and RNA isolation using the solution-based approach from *Thymus vulgaris* and *Malus domestica*; (b) concentration and purity of nucleic acid isolated from various plant species (*Malus domestica*, *Pyrus communis* L, *Brassica napus*, *Hordeum vulgare*); (c) RNA isolation using the column-based approach from cotton pollen tetrads (*G*. *hirsutum*) shown in three replicates (TE R1, TE R2 and TE R3); (d) concentration and integrity of RNA isolated from cotton pollen tetrads (*G*. *hirsutum*) shown in three replicates (TE R1, TE R2 and TE R3) and an electropherogram from TE R1; (e) DNA isolation using the column-based approach from cotton leaf (*G*. *robinsonii*) shown in two replicates (Leaf R1 and Leaf R2); (f) concentration and purity of nucleic acid isolated from cotton leaf shown in two replicates (Leaf R1 and Leaf R2).

Fig 2 demonstrates the quality and quantity of nucleic acid isolation using the solution-based (Fig 2A and 2B) and column-based (Fig 2C–2F) approaches. As shown in Fig 2A, the solution-based approach resulted in the simultaneous isolation of DNA and RNA from *Thymus vulgaris* and *Malus domestica*. It contained genomic DNA fragments of around 20,000 bp for these two species and intact total RNA with sharp 28S and 18S ribosomal RNA (rRNA) bands, demonstrating high-quality DNA and RNA isolated from the same tissue. This approach led to a high concentration of nucleic acid varying between 271.8 (*Brassica napus*) and 1335.8 (*Malus domestica*) ng/μl, measured by Nanodrop (Thermo Fisher Scientific), depending on the species. The isolated DNA and RNA provided a 260/280 nm absorbance ratio of approximately 2 and a 260/230 nm absorbance ratio of 1.5–2.3, demonstrating the purity of nucleic acid, which is ideal for downstream molecular analysis (Fig 2B).

The lysis buffer pH (Fig 1, Step 1) is approximately 8.5–9, simultaneously isolating DNA and RNA from plant tissues. Reducing the pH level of the lysis buffer to about 6–7 enables the isolation of only RNA from plant tissues as the acidic lysis buffer precipitates DNA into the organic phase. Alternatively, the isolated DNA and RNA from plant tissues can be treated with either DNase I or RNase I, according to the manufacturer’s instructions, to obtain pure RNA and DNA, respectively.

The column-based approach for DNA or RNA isolation is highly recommended for NGS applications, such as WGS and RNA-seq analyses. This approach resulted in high-quality RNA isolated from cotton pollen tetrads, shown in three replicates (TE R1, TE R2 and TE R3; Fig 2C, 2D). The results demonstrated the suitability of the RNA to construct a library for RNA-seq analysis using the Illumina NovaSeq 6000 platform [18]. The quality of RNA isolated using the protocol was measured by Epoch spectrophotometer (BioTek, Winooski, VT, USA) and Agilent TapeStation (Agilent Technologies, Santa Clara, CA, USA), demonstrating intact total RNA, sharp 28S and 18S rRNA bands, a distinct electropherogram of rRNA, sufficient concentration, a 28S/18S ratio of about 2:1 and high RNA integrity number (RIN).

The column-based approach was also used to isolate high-quality DNA from leaf tissues of *G*. *robinsonii* (wild cotton species). Fig 2E and 2F shows the outcome of DNA isolation from the wild cotton leaves, shown in two replicates (Leaf R1 and R2). The genomic DNA fragments were intact, shown on the gel, and around 23,000 bp for *G*. *robinsonii*. A fluorometer (Qubit, Invitrogen) was used to measure the concentration of DNA samples, confirming the appropriate concentration of the isolated DNA for WGS analysis [17].

Overall, the modified CTAB method described here isolates high-quality DNA from various plant species. Surprisingly, the protocol also isolates RNA from the same tissues, unlike previously modified CTAB methods, which makes it an ideal method to perform various molecular analyses. Moreover, the modified CTAB method is effective for small amounts of plant tissues when collecting adequate samples is a limiting factor, such as the developmental stages of pollen (e.g., tetrads). The simple approach introduced in the protocol enables handling many samples in a timely manner, which might be essential for extensive laboratory experiments. Importantly, the protocol is accompanied by silica-membrane columns to purify high-quality DNA or RNA, which is recommended for high-throughput sequencing platforms. The modified CTAB protocol described here can provide a great opportunity for plant researchers to perform molecular analyses from various plant species, including recalcitrant plants.

## Supporting information

S1 File Step-by-step protocol, also available on protocols.io. (PDF)
