# Supplementary material for: A universal protocol for high-quality DNA and RNA isolation from diverse plant species
Source: PLoS One. 2023 Dec 14;18(12):e0295852. doi: 10.1371/journal.pone.0295852 (PMC10721051; doi:10.1371/journal.pone.0295852)
Supplement: S1 File — (PDF) [file pone.0295852.s001.pdf]

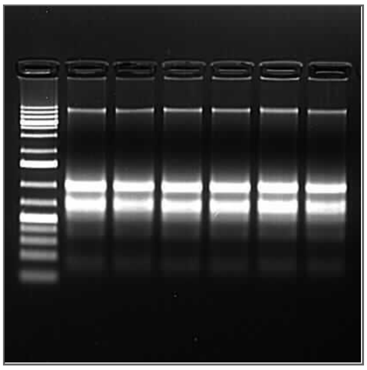

VERSION 3

DEC 05, 2023

OPEN ACCESS

DOI:
dx.doi.org/10.17504/protocols.io.8epv5xj86g1b/v3

Protocol Citation: Farhad Masoomi-Aladizgeh, Leila Jabbari, Reza Khayam Nekouei, Ali Aalami, Brian J Atwell, Paul A Haynes 2023. A universal protocol for high-quality DNA and RNA isolation from diverse plant species. **protocols.io** https://dx.doi.org/10.17504/protocols.io.8epv5xj86g1b/v3Version created by Farhad Masoomi-Aladizgeh

MANUSCRIPT CITATION:

A universal protocol for high-quality DNA and RNA isolation from diverse plant species V.3

Farhad Masoomi-Aladizgeh[1], Leila Jabbari[2], Reza Khayam Nekouei[3], Ali Aalami[3], Brian J Atwell[1], Paul A Haynes[1]

[1]School of Natural Sciences, Macquarie University, NSW, Australia;
[2]Department of Tissue Culture and Gene Transformation, Agricultural Biotechnology Research Institute of Iran (ABRII), AREEO, Karaj, Iran;
[3]Department of Agronomy and Plant Breeding, Faculty of Agricultural Sciences, University of Guilan, Rasht, Iran

Nucleic Acid Isolation

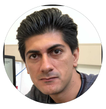

Farhad Masoomi-Aladizgeh
Macquarie University

ABSTRACT

Next-generation sequencing demands high-quality nucleic acid, yet isolating DNA and RNA from plant tissues is often challenging. Despite advancements in the development of a variety of kits and reagents, these products are only limited to isolating nucleic acid from model plant species. In this study, a universal lysis buffer is introduced to isolate nucleic acid from a wide range of plant species, including recalcitrant plants, to facilitate molecular studies, such as quantitative PCR (qPCR), transcriptomics, whole-genome sequencing, etc. The lysis buffer consists of hexadecyltrimethylammonium bromide (CTAB), sodium chloride (NaCl), Tris base, ethylenediaminetetraacetic acid (EDTA) and β-mercaptoethanol (βME). The appropriate concentration of the components creates an ideal pH, which then facilitates the isolation of DNA and RNA from plant tissues simultaneously. This is one of the key differences between this protocol and other CTAB protocols that use similar reagents but have been optimised to isolate only DNA. For DNA and RNA to be qualified for next-generation sequencing platforms, the protocol is supplemented with columns to purify either DNA or RNA from the same tissue to meet high standards for sequencing analyses. This protocol provides an ideal approach to overcome potential obstacles in isolating high-quality DNA or RNA from a wide range of plant species in a timely manner. It is well-suited for a large number of samples and also when adequate sample collection is a limiting factor.

License: This is an open access protocol distributed under the terms of the Creative Commons Attribution License, which permits unrestricted use, distribution, and reproduction in any medium, provided the original author and source are credited

Protocol status: Working
We use this protocol and it's working

Created: Dec 05, 2023

Last Modified: Dec 05, 2023

PROTOCOL integer ID: 91869

Keywords: DNA isolation, Next generation sequencing, Plants, Recalcitrant species, RNA isolation

MATERIALS

REAGENTS

- Hexadecyltrimethylammonium bromide (CTAB; Sigma-Aldrich, CAS 57-09-0)
- Tris(hydroxymethyl)aminomethane (Tris base; Sigma-Aldrich, CAS 77-86-1)
- Ethylenediaminetetraacetic acid (EDTA; Sigma-Aldrich, CAS 60-00-4)
- Sodium chloride (NaCl; Astral Scientific, CAS 7647-14-5)
- β-mercaptoethanol (βME; Sigma-Aldrich, CAS 60-24-2)
- Chloroform (Sigma-Aldrich, CAS 67-66-3)
- Ethanol (Chem-Supply Pty Ltd Australia, CAS 64-17-5)
- DNeasy Plant Pro Kit (Qiagen, CAT 69204)
- DNeasy Plant Mini Kit (Qiagen, CAT 74904)

EQUIPMENT

- NanoDrop spectrophotometer (Thermo Fisher Scientific, CAT ND-2000)
- Micropipetts (Eppendorf, 0.5 – 10 µL, 10 – 100 µL, 100 – 1,000 µL single-channel, variable pipettes)
- Microcentrifuge Tube, Sterile (SSIBio, 2 mL, 1.5 mL)
- Mortar and pestle
- TissueLyser II (Qiagen, CAT 85300)
- Water bath (Thermoline Scientific)
- Chemical fume hood (Dynaflow)
- Centrifuge (Eppendorf, 5424 R)
- Gel Doc and electrophoresis (BioRad)
- PH meter (Hanna Instruments, HI11310)

Lysis Buffer for DNA and RNA Isolation

1 The lysis buffer contains 0.5% CTAB, 1% EDTA, 2.5% Tris base and 5% NaCl. These are the four main components of the lysis buffer for DNA and RNA isolation from plant tissues.
Example: Add CTAB (125 mg), EDTA (250 mg), Tris base (625 mg), and NaCl (1250 mg) to 25 ml nuclease-free water, and then dissolve the components in the given order by shaking at room temperature.
CRITICAL STEP 1: PH adjustment of the lysis buffer
Option 1: The lysis buffer's pH is already 8.5-9, simultaneously isolating DNA and RNA from the mixture.
Option 2: Reducing the pH level of the mixture to 6-7 enables the isolation of only RNA from the mixture. The acidic lysis buffer precipitates DNA into the organic phase.

CRITICAL STEP 2: Supplements to the lysis buffer
β-mercaptoethanol (βME): For DNA and RNA isolation, add 50 µl (5%) βME to 1 ml of the lysis buffer before use to decrease the probable oxidation only for tissues with high polysaccharides and secondary metabolites. βME is highly recommended for RNA isolation since it eliminates RNAses

released during cell lysis.
Polyvinylpolypyrrolidone (PVPP): For DNA isolation, add 15 mg (1.5%) PVPP per 1 ml of the lysis buffer only for tissues with high polyphenolic compounds to increase the concentration of DNA.

Homogenization of Tissues

2 Grind plant tissues (leaf, shoot, root, etc., approximately 0.1 g) following one of the below options:
Option 1: Grind the tissues with 1 ml of the lysis buffer in a sterilized mortar and pestle without liquid nitrogen.
Option 2: Grind the tissues with a TissueLyser using up to six Zirconox beads (2.8–3.3 mm) and liquid nitrogen. Then, mix the ground tissue with 1 ml of the lysis buffer, followed by vigorous vortexing to create a homogeneous mixture.
CRITICAL STEP: βME can be added to the homogenised plant tissues at this stage. Alternatively, it can be added directly to the lysis buffer as described in the first section. The former is recommended as it can be easily managed inside a chemical fume hood.

Triple-Phase Separation

3 Incubate samples at 65°C for 10 min to lyse cells completely. Invert the mixture several times during the incubation time or use a thermo shaker incubator with constant shaking at 300 rpm.
Add 600 µl chloroform to each sample and homogenize the mixture by vortexing vigorously, followed by centrifugation at 13,700 *g* at 4°C for 10 min.
The centrifugation of the mixture results in three phases, as shown in Figure 1:

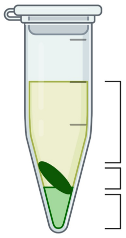

Figure 1. Triple-phase separation using chloroform

Transfer the upper aqueous phase (600 µl) to a new 2 ml microcentrifuge tube gently, without disrupting the solid and organic phases. This phase can undergo solution-based or column-based approaches for nucleic acid purification and precipitation.
CRITICAL STEP 1: The upper aqueous phase with the isolation buffer at pH 8.5-9 consists of DNA and RNA, while the isolation buffer at pH 6-7 consists of RNA since DNA precipitates at an acidic pH.
CRITICAL STEP 2: Continue the remaining steps at cool conditions, handling samples on ice and centrifugation at 4°C, only if RNA isolation is considered.

Purification and Precipitation of Nucleic Acid (solution-based...

4 **The following procedure describes a solution-based approach for the purification and precipitation of nucleic acid, which is recommended for routine PCR, qPCR and Sanger sequencing applications.**

Collect the upper aqueous phase (600 µl), add 1.5 volume (900 µl) of cold isopropanol to the mixture and invert the tube gently to mix the solution. A stringy white mass might be visible at this stage, representing nucleic acids.
Centrifuge the samples at 13,700 *g* at 4°C for 10 min. The white pellet will be visible on the bottom of the tubes.
Discard the supernatant and wash the pellet gently with 70% EtOH.
Centrifuge the samples at 5400 *g* at 4°C for 5 min, remove the supernatant and then air dry the pellet.
Dissolve the pellet in 30 µl of RNase-free or autoclaved water by incubating at room temperature. Dissolving the pellet should take only a few minutes if the extracted DNA or RNA is pure.
DNAase I and RNase I can be used to eliminate DNA and RNA, respectively, according to the manufacturer's instructions.

Purification of and Precipitation Nucleic Acid (column-based a...

5 **The following procedure describes a column-based approach for the purification and precipitation of nucleic acid, which is recommended for Next Generation Sequencing applications, such as DNA-seq or RNA-seq.**
DNA isolation (coupled with the Qiagen DNeasy Plant Mini Kit):
Collect the upper aqueous phase (600 µl), add 1.5 volume of buffer AW1 to the lysate, and mix it immediately by pipetting. Do not centrifuge the lysate at this stage. For example, to 600 µl supernatant, add 900 µl buffer AW1.
Transfer the mixture (~ 600 µl each time) into a **DNeasy Mini spin column (white)**, including any precipitate. Centrifuge the samples at 8,000 *g* for 30 s, and discard the flow-through. Repeat the step with the remaining lysate.
Place the DNeasy Mini spin column into a new 2 ml microcentrifuge tube, add 500 µl buffer AW2 and centrifuge at 8,000 *g* for 30 s. This step may be repeated more than once, especially if the spin column membrane is not clean.
Transfer the DNeasy Mini spin column to a 1.5 ml microcentrifuge tube, and pipet 30 µl RNAse-free water directly onto the DNeasy membrane. After about 5 minutes, centrifuge at 5,000 *g* for 2 min to elute DNA.
To eliminate any remaining RNA, treat the samples with 2 µl RNase I at room temperature for 10 min.
RNA isolation (coupled with the Qiagen RNeasy Plant Mini Kit):
Collect the upper aqueous phase (600 µl), add 0.5 volume of ethanol (100%) to the lysate, and mix it immediately by pipetting. Do not centrifuge the lysate at this stage. For example, to 600 µl supernatant, add 300 µl ethanol.
Transfer the mixture (~ 600 µl each time) into an **RNeasy spin column (pink)**, including any precipitate. Centrifuge the samples at 8,000 *g* for 30 s, and discard the flow-through. Repeat the step with the remaining lysate.
Place the RNeasy spin column into a new 2 ml microcentrifuge tube, add 700 µl buffer RW1 and centrifuge at 8,000 *g* for 30 s.
Add 500 µl buffer RPE to the RNeasy spin column and centrifuge at 8,000 *g* for 30 s to wash the spin column membrane (ensure that ethanol is added to the buffer). This step may be repeated more than once, especially if the spin column membrane is not clean.
Transfer the RNeasy spin column to a 1.5 ml microcentrifuge tube, and pipet 30 µl RNAse-free water directly onto the RNeasy membrane. After about 5 minutes, centrifuge at 5,000 *g* for 2 min to elute

RNA.
To eliminate any remaining DNA in the samples, treat the samples with DNAase I according to the manufacturer's instructions.

Storage Condition

6 Nucleic acids can be stored either at −20°C for short time storage or at −80°C for longtime storage.

Workflow for Nucleic Acid Isolation

7

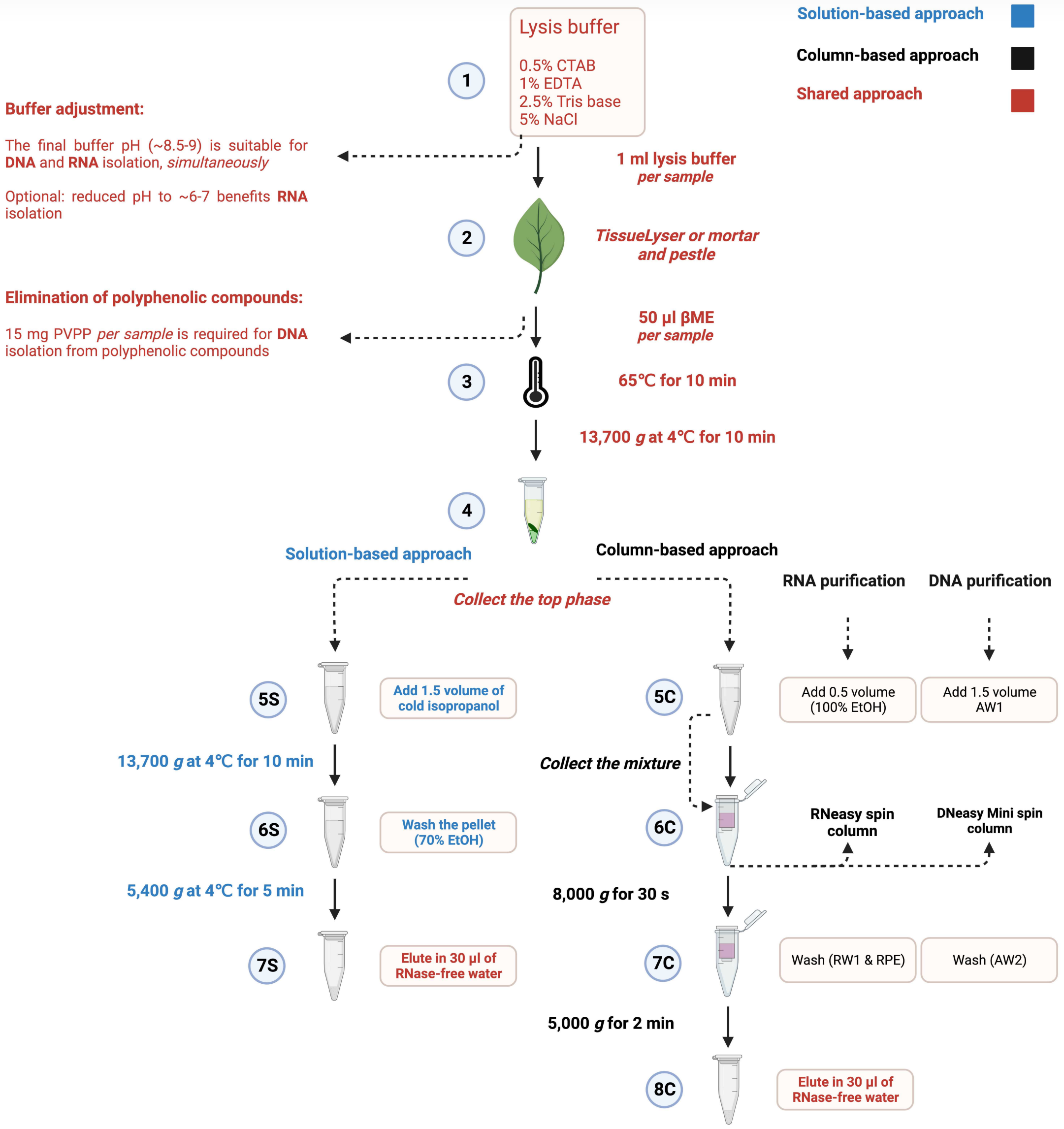

Figure 2. Nucleic acid isolation from plant species. Steps 1-4 include the preparation of lysis buffer for DNA and RNA Isolation (Step 1), homogenization of tissues (Step 2), lysis of tissues (Step 3) and triple-phase separation (Step 4), the main steps of nucleic acid isolation. **Optional step:** Reduce the pH of the lysis buffer to 6-7 to isolate only RNA. The upper phase, containing nucleic acid collected from Step 4, can be purified either using the solution-based approach (5S-7S) or the column-based approach (5C-8C). **Solution-based**

approach: Collect the upper phase from Step 4 and mix it with 1.5 volume of cold isopropanol (Step 5S). Centrifuge the mixture at 13,700 *g* at 4°C for 10 min. Discard the supernatant and wash the pellet gently with 70% EtOH, followed by centrifugation at 5400 *g* at 4˚C for 5 min (Step 6S). Remove the supernatant, and then air dry the pellet. Resuspend the pellet in 30 µl of RNase-free water by incubating at room temperature (Step 7S). **Column-based approach for DNA:** Collect the upper phase from Step 4, add 1.5 volume of buffer AW1 to the lysate, and mix it immediately by pipetting (Step 5C). Transfer the mixture into a DNeasy Mini spin column (white), including any precipitate. Centrifuge the samples at 8,000 *g* for 30 s, and discard the flow-through (Step 6C). Place the DNeasy Mini spin column into a new tube, add 500 µl buffer AW2 and centrifuge at 8,000 *g* for 30 s (Step 7C). Transfer the DNeasy Mini spin column into a new tube, and pipet 30 µl RNAse-free water directly onto the DNeasy membrane. Centrifuge the samples at 5,000 *g* for 2 min, after about 5 minutes, to elute DNA (Step 8C). **Column-based approach for RNA:** Collect the upper phase from Step 4, add 0.5 volume of ethanol (100%) to the lysate, and mix it immediately by pipetting (Step 5C). Transfer the mixture into an RNeasy spin column (pink), including any precipitate. Centrifuge the samples at 8,000 *g* for 30 s, and discard the flow-through (Step 6C). Place the RNeasy spin column into a new tube, add 700 µl buffer RW1 and centrifuge at 8,000 *g* for 30 s. Add 500 µl buffer RPE to the RNeasy spin column and centrifuge at 8,000 *g* for 30 s to wash the spin column membrane (Step 7C). Transfer the RNeasy spin column to a new tube, and pipet 30 µl RNAse-free water directly onto the RNeasy membrane. Centrifuge the samples at 5,000 *g* for 2 min, after about 5 minutes, to elute RNA (Step 8C).

Expected Results

8

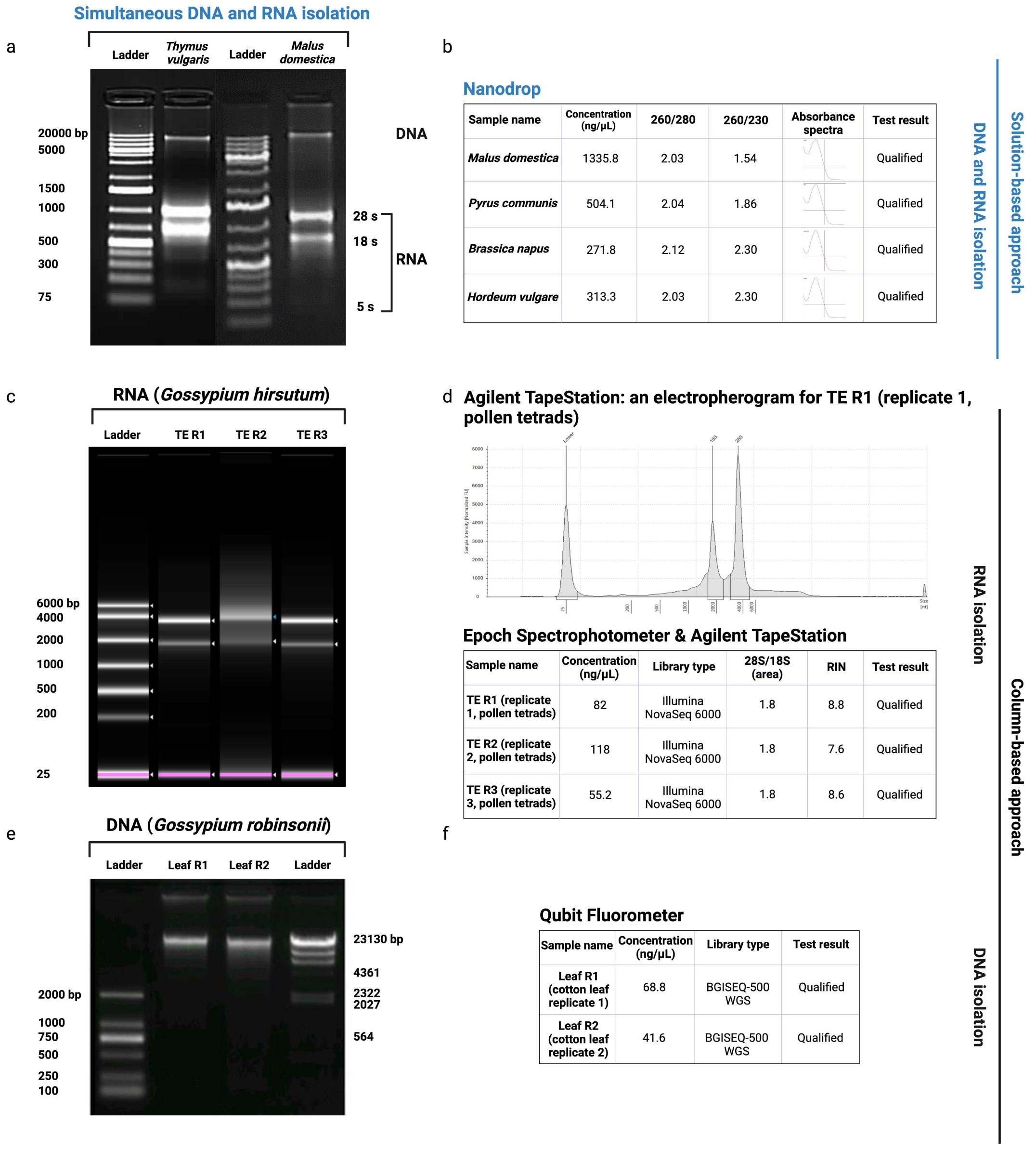

Sample name	Concentration (ng/μL)	260/280	260/230	Absorbance spectra	Test result
Malus domestica	1335.8	2.03	1.54		Qualified
Pyrus communis	504.1	2.04	1.86		Qualified
Brassica napus	271.8	2.12	2.30		Qualified
Hordeum vulgare	313.3	2.03	2.30		Qualified

Sample name	Concentration (ng/μL)	Library type	28S/18S (area)	RIN	Test result
TE R1 (replicate 1, pollen tetrads)	82	Illumina NovaSeq 6000	1.8	8.8	Qualified
TE R2 (replicate 2, pollen tetrads)	118	Illumina NovaSeq 6000	1.8	7.6	Qualified
TE R3 (replicate 3, pollen tetrads)	55.2	Illumina NovaSeq 6000	1.8	8.6	Qualified

Sample name	Concentration (ng/μL)	Library type	Test result
Leaf R1 (cotton leaf replicate 1)	68.8	BGISEQ-500 WGS	Qualified
Leaf R2 (cotton leaf replicate 2)	41.6	BGISEQ-500 WGS	Qualified

Figure 3. Quality and quantity of nucleic acid isolation using solution-based and column-based approaches. (a) **DNA and RNA** isolation using the **solution-based approach** from *Thymus vulgaris* and *Malus domestica*; (b) concentration and purity of nucleic acid isolated from various plant species (*Malus domestica*, *Pyrus communis* L, *Brassica napus*, *Hordeum vulgare*); (c) **RNA** isolation using the **column-based approach** from cotton pollen tetrads (*Gossypium hirsutum*) shown in three replicates (TE R1, TE R2 and TE R3); (d) concentration and integrity of RNA isolated from cotton pollen tetrads (*Gossypium hirsutum*) shown in three replicates (TE R1, TE R2 and TE R3) and an electropherogram from TE R1; (e) **DNA** isolation using the **column-based approach** from cotton leaf (*Gossypium robinsonii*) shown in two replicates (Leaf R1 and Leaf R2); (f) concentration and purity of nucleic acid isolated from cotton leaf shown in two replicates (Leaf R1 and Leaf R2).
